# Digital Therapeutics (DTx) Expand Multimodal Treatment Options for Chronic Low Back Pain: The Nexus of Precision Medicine, Patient Education, and Public Health

**DOI:** 10.3390/healthcare11101469

**Published:** 2023-05-18

**Authors:** Aarushi Rohaj, Grzegorz Bulaj

**Affiliations:** 1 The Spencer Fox Eccles School of Medicine, University of Utah, Salt Lake City, UT 84112, USA; 2 Department of Medicinal Chemistry, L.S. Skaggs College of Pharmacy, University of Utah, Salt Lake City, UT 84112, USA

**Keywords:** digital health, neurological disorders, chronic pain, mHealth, smartphone, self-management, analgesics, drug-digital combination, pain management

## Abstract

Digital therapeutics (DTx, software as a medical device) provide personalized treatments for chronic diseases and expand precision medicine beyond pharmacogenomics-based pharmacotherapies. In this perspective article, we describe how DTx for chronic low back pain (CLBP) can be integrated with pharmaceutical drugs (e.g., NSAIDs, opioids), physical therapy (PT), cognitive behavioral therapy (CBT), and patient empowerment. An example of an FDA-authorized DTx for CLBP is RelieVRx, a prescription virtual reality (VR) app that reduces pain severity as an adjunct treatment for moderate to severe low back pain. RelieVRx is an immersive VR system that delivers at-home pain management modalities, including relaxation, self-awareness, pain distraction, guided breathing, and patient education. The mechanism of action of DTx is aligned with recommendations from the American College of Physicians to use non-pharmacological modalities as the first-line therapy for CLBP. Herein, we discuss how DTx can provide multimodal therapy options integrating conventional treatments with exposome-responsive, just-in-time adaptive interventions (JITAI). Given the flexibility of software-based therapies to accommodate diverse digital content, we also suggest that music-induced analgesia can increase the clinical effectiveness of digital interventions for chronic pain. DTx offers opportunities to simultaneously address the chronic pain crisis and opioid epidemic while supporting patients and healthcare providers to improve therapy outcomes.

## 1. Introduction

Increasing the prevalence of chronic pain and opioid epidemic are examples of public health challenges that negatively impact individual lives and healthcare systems. Worldwide, 577 million people experienced low back pain in 2017 [1]. According to the Center for Disease Control and Prevention (CDC), 39% of adults in the US had low back pain in 2019 [2]. Chronic low back pain (CLBP) is a painful neurological disorder that affects the lower segment of the spine [3]. CLBP is characterized as pain that occurs consistently for 12 weeks or longer and is one of top causes of disability around the world [4], including the leading cause of years lived with disability [1]. CLBP patients experience reduced health-related quality of life (HRQoL) [5] and suffer from comorbidities such as sleep disorders, anxiety, and depression [6,7,8,9,10,11,12]. People living with CLBP report a negative impact of the disorder on their personal relationships, social life, and work [13].

**Figure 1:**
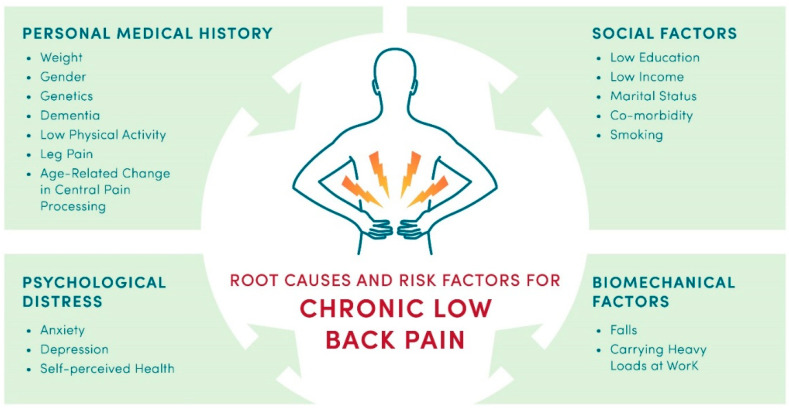
Root causes and risk factors for CLBP.

A majority of CLBP causes are nonspecific and idiopathic, or mechanical (spinal stenosis, radiculopathy, traumatic injury and/or overuse of the spine). Other root causes of CLBP include biomechanical factors such as carrying heavy loads at work and personal medical history, such as higher body mass index [14]. Figure 1 shows examples of modifiable and non-modifiable risk factors that lead to CLBP [14,15,16,17], and also can cause transition from acute low back pain to CLBP [14,17,18,19,20]. Modifiable risk factors include sedentary lifestyle, smoking, falls, social environment, and self-perceived health. Non-modifiable risk factors include genetics, age, family history, and dementia, among others. It is noteworthy that adverse childhood experiences can also increase a risk for CLBP [21,22]. A complex combination of causes and psychosocial, cognitive, and biomechanical factors impacts both the intensity of CLBP and treatment options.

Digital health technologies comprise digital therapeutics (mobile medical apps), mobile health (mHealth) apps, and telemedicine to improve patient care. This relatively new area of health care has shown promise for improving therapy outcomes in diverse chronic diseases ranging from diabetes [23] and depression [24] to insomnia [25] and pain [26]. VR and augmented reality technologies are gaining recognition as a means to improve pain relief [27], mental health [28], neurorehabilitation [29], and physical therapy [30]. Mobile medical apps (digital therapeutics) intended to treat specific medical conditions are regulated by the Food and Drug Administration as medical devices (software as a medical device, SaMD) [31,32,33,34]. There are several VR and mobile medical apps that received market authorization from the FDA as either non-prescription or prescription digital therapeutics (PDT) to treat chronic diseases such as addiction, ADHD, insomnia, diabetes, and CLBP [32,35]. In contrast to non-prescription DTx (also known as over-the-counter or OTC DTx), PDTs are available to patients only by prescription from healthcare professionals [36,37].

The main objectives of this perspective article are: (1) to provide an overview of the current therapies used to treat CLBP in the context of emerging digital therapeutics (DTx), (2) to describe opportunities of DTx to integrate multimodal treatment options for CLBP, (3) to discuss advances of DTx towards exposome-responsive, just-in-time digital interventions, and (4) to encourage all health care stakeholders to advocate for research, development and implementation of DTx to improve therapy outcomes and public health. We hope that this work will contribute towards a broader and deeper understanding of opportunities for DTx to expand multimodal treatment options for neurological disorders.

## 2. Current Non-Pharmacological and Pharmacological Treatments for CLBP

**Figure 2:**
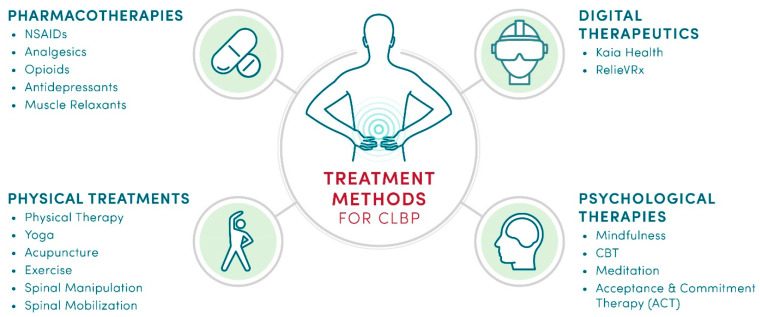
The diversity of non-pharmacological and pharmacological treatments for CLBP.

Given the complexity of the causes of CLBP, there are many diverse treatments addressing both etiology and symptoms [38,39,40,41,42,43,44,45,46]. As illustrated in Figure 2, non-pharmacological interventions include physical and psychological therapies, as well as digital therapies that can deliver both. Depending on the spinal injury, surgical solutions, such as spinal fusion or disc replacement, may offer clinical benefits [47]. Another example of non-pharmacological treatment of CLBP is transcutaneous electrical nerve stimulation (TENS) [48]. Spinal cord stimulation [49,50,51] and nerve ablation [52,53] are also evaluated for their effectiveness in treating CLBP. In addition to diverse therapies and surgeries, self-management and patient education have been recognized as viable means to reduce pain intensity in CLBP patients [54,55,56,57].

Physical therapy is effective in improving pain and reducing disability in CLBP patients [58,59]. Recent network meta-analyses reported that among diverse physical exercise options, Pilates, core strengthening, and McKenzie and functional restoration methods, appeared to be the most effective in improving pain intensity [60,61], while yoga, acupuncture, and spinal manipulation can also reduce CLBP [61,62,63,64,65]. Psychological therapies such as cognitive behavioral therapy (CBT), mindfulness meditation and mindfulness-based stress reduction can improve pain, physical functions, and health-related quality of life of CLBP patients [66,67,68,69,70].

Pharmacological treatments of CLBP include analgesics, muscle relaxants, antidepressants, and anticonvulsant drugs [71,72,73,74,75,76,77,78,79]. Acetaminophen and NSAIDs (ibuprofen and naproxen) are commonly used as either over-the-counter (OTC) or prescription analgesics for CLBP [42]. While opioid-based analgesics provide a short-term reduction in CLPB, their long-term use is controversial due to adverse effects [80,81]. Over-prescription of opioid analgesics for pain resulted in the opioid epidemic crisis in North America [82,83,84,85]. A recent network meta-analysis suggested that only NSAIDs and opioids are effective in improving both pain and functions in CLPB patients [41]. While pharmacological interventions can offer pain relief, limitations of medications for CLBP are drug–drug interactions [86], gastrointestinal and cardiovascular toxicities [87], and the development of opioid use disorder [88].

**Figure 3:**
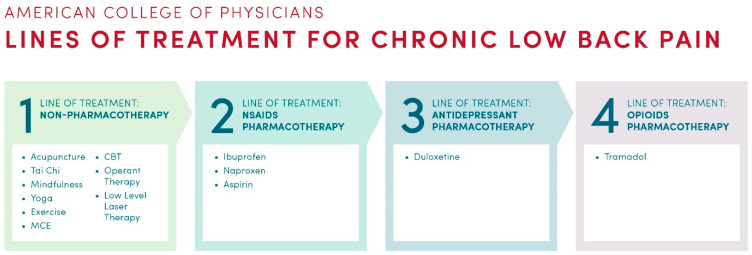
The clinical practice guidelines from the American College of Physicians recommending treatments for CLBP.

In 2017, the American College of Physicians (ACP) published evidence-based clinical guidelines for the treatment of CLBP [39]. As shown in Figure 3, ACP recommends that CLBP patients should initially start with non-pharmacological treatments [39]. Patients that have an inadequate response to the non-drug therapies should use NSAIDs as a second line of treatment. The next lines of recommended pharmacotherapies are duloxetine (antidepressant) and then tramadol (opioid). While opioids should be considered as the last option for treatment, they are widely used by CLBP patients [75], in particular those with severe pain [13]. It is noteworthy that many non-pharmacological treatments for CLBP recommended in the ACP guidelines are not covered by insurance as essential health benefits [89].

To address both the complexity of etiology and the diversity of risk factors for CLBP, the multimodal approach offers apparent advantages over monotherapies. Combining physical and psychological therapies for CLBP offer additional benefits; for example, PT and CBT or ACT can further improve pain relief and disability as compared to PT alone [90,91,92]. A multidisciplinary 8-week restoration program for CLBP patients was created by combining physical and psychological interventions (functional strength, calisthenics, aerobics, stretching, education, socioeconomic counseling, and CBT) [93]. Based on RCT, the program decreased the use of medical treatment; 42% of the patients refrained from taking analgesics, and 63% of the patients returned to active/productive work after the program [93].

The multimodal approach to treat CLBP has been recognized by The Back Pain Research Consortium, the National Institute of Health (NIH) initiative to mitigate the opioid crisis, so-called “Helping to End Addiction Long-term” or HEAL [38]. This group has proposed to develop a “precision medicine algorithm” based on combining three non-pharmacological (exercise and manual therapies, ACT and self-management) and one pharmacological (duloxetine) treatment [38]. Commonly used multimodal combinations for CLBP include physical therapy and analgesics or psychotherapies and analgesics.

## 3. Emergence of Digital Therapeutics for CLBP

Digital health technologies for CLBP provide diverse treatment and self-management modalities [35,55,94,95,96,97,98,99,100,101,102,103,104,105,106,107,108]. Examples of mobile apps for CLBP include “BackRx” delivering yoga and Pilates exercises for patients with discogenic CLPB [97,109], as well as PainNavigator delivering CLBP management, including education, physical exercises, yoga, and mindfulness [110]. A 12-week study of the use of a mobile app delivering the digital care program Hinge Health for low back pain reported improvement in pain and disability outcomes, as well as depression and anxiety symptoms [111,112]. The “Active ingredients” of this digital care program included exercise therapy, CBT, education, behavioral coaching, and tracking activity and symptoms [111]. Cost-effectiveness analysis of digital interventions for CLBP was favorable for the VR-based therapy, as compared to clinic-based McKenzie method [113].

Another example of digital therapy for back pain is the Kaia Health app that delivers multimodal self-management content [114,115,116]. The Kaia Back Pain app guides users through physical exercises, patient education and relaxation techniques (breathing exercises and progressive muscle relaxation), using a computer vision motion analysis that enables patient’s spatial awareness, ensures proper form during exercise, and quantifies movement as a digital biomarker. The Kaia Back Pain app is intended for adults with non-specific back pain that persists for 4 weeks or longer and is marketed in the US as a low-risk medical device under the FDA enforcement discretion. In Europe, it has the status of class IIa medical device.

RelieVRx (earlier known as EaseVRx) is a prescription digital therapeutic (PDT), an adjunct treatment for CLBP, which received market authorization from the FDA in November 2021 [35]. This digital intervention is available only by prescription from healthcare providers. The SaMD regulatory status of RelieVRx was granted via the 510 k, De Novo premarket pathway, while earlier RelieVRx received the Breakthrough Device designation from the FDA. RelieVRx is an immersive VR system delivering multimodal therapeutic content to treat moderate to severe CLBP in adult patients.

**Figure 4:**
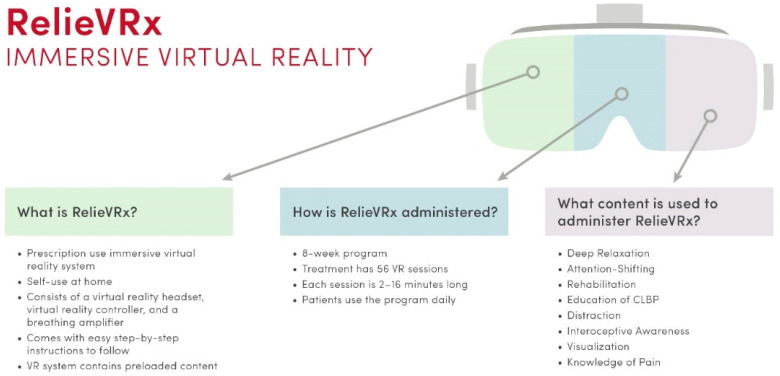
RelieVRx is a prescription digital therapeutic (PDT) for CLBP, delivering multimodal therapeutic content via VR-based technology.

**Figure 5:**
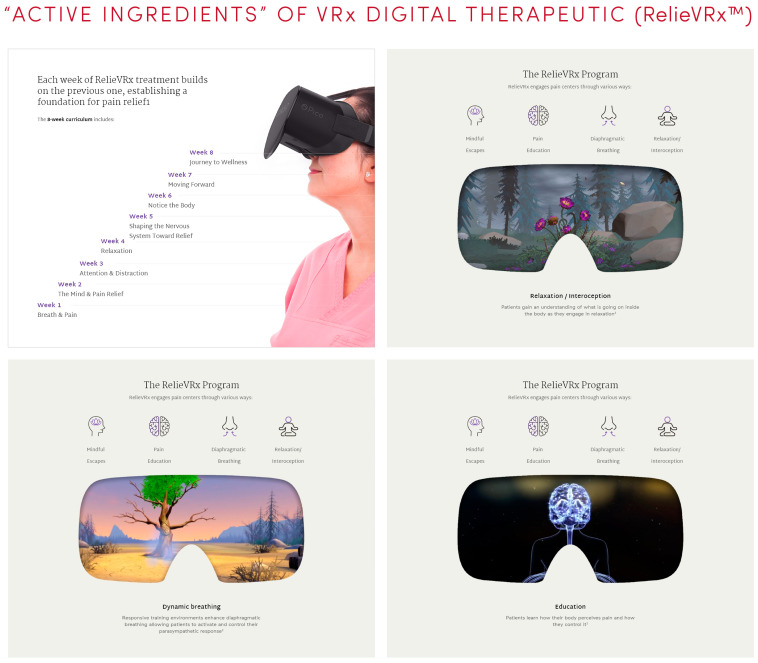
The digital therapy content of RelieVRx. Images show weekly themes and screenshots of what CLBP patients see and experience using the RelieVRx system. Images are courtesy of AppliedVR and are available on their website (https://www.appliedvr.io/).

As illustrated in Figure 4 and Figure 5, the multimodal content of RelieVRx includes diverse behavioral and CBT-based “active ingredients” intended to reduce pain, namely patient education, deep relaxation, interoceptive awareness, attention-shifting, distraction, immersive enjoyment, and acceptance of CLBP. Combined together, these aforementioned elements are used to train the patient’s brain (e.g., executive, emotional, and multisensory pathways) to think differently about how it experiences pain. The digital therapy consists of 56 VR sessions, each lasting 2–16 min and are used as part of the daily eight-week program. Each session incorporates the different principles mentioned above to achieve pain relief and reduction in pain interference in daily activities. RelieVRx system consists of a virtual reality controller, headset, and a breathing amplifier that is attached to the headset, which helps in directing a patient’s breath during deep breathing exercises. The system with instructions is shipped to a patient’s home and then returned after the 8-week treatment.

RelieVRx was evaluated in CLBP patients in a 21-day pilot RCT [117], as well as in a 56-day, double-blind, placebo-controlled RCT [98,117,118,119,120]. The pivotal efficacy and safety RCT of RelieVRx (registered as NCT00415177 in clinicaltrials.gov) included 179 CLBP patients assigned to either the eight-week treatment group using RelieVRx 3D immersive pain relief program, or to the control group (sham VR program displayed 2D nature content and did not provide skills training) [118]. Pain relief and other outcomes were measured using a Defense and Veterans Pain Rating Scale which measured pain intensity, and interference around a patient’s activities, sleep, mood, and stress. Additional measures were collected using the NIH Physical Function and Sleep Disturbance form, Pain Catastrophizing Scale, Pain Self-Efficacy Questionnaire, and Chronic Pain Acceptance. To evaluate the quality of life, the self-reported Patient’s Global Impression of Change Scale was used. Follow-up data from the pivotal RCT were collected and reported after 3, 6, and 18 months [98,119,120].

At the end of the eight weeks, the RelieVRx participants experienced a significant pain intensity reduction of an average of 42.8%, as compared to 25.1% for the control group (*p* < 0.001), while 46% of patients in the treatment group reached 50% or more pain reduction, as compared to 26% patients in the control group [118]. The treatment group also reported a significant reduction in sleep disturbance, pain interference (mood, stress, and activity), and physical function, as compared to the control VR program. Based on the safety evaluation of RelieVRx, no participants contacted the staff to report discomfort with the virtual reality headset or experienced any motion sickness and nausea. During the 3-month follow-up, the RelieVRx participants reported 30.3% mean pain intensity improvement, while for the control participants this value was 15.8% [98]. Participants in the treatment VR program maintained significant improvement in clinical outcomes after six months, as summarized by the investigators: “Therapeutic VR maintained significant and clinically meaningful effects 6 months posttreatment and remained superior to sham VR for reducing pain intensity and pain-related interference with activity, stress, and sleep (*d_s_* = 0.44–0.54; *p* < 0.003)” [119]. Positive outcomes were also observed after 18 months posttreatment [120]. An additional RCT is intended to evaluate therapy outcomes among CLBP patients such as physical functions, sleep, anxiety, depression, and opioid-based pain management [98,117,118,119,120,121].

## 4. DTx Expand Multimodal Therapies for CLBP

**Figure 6:**
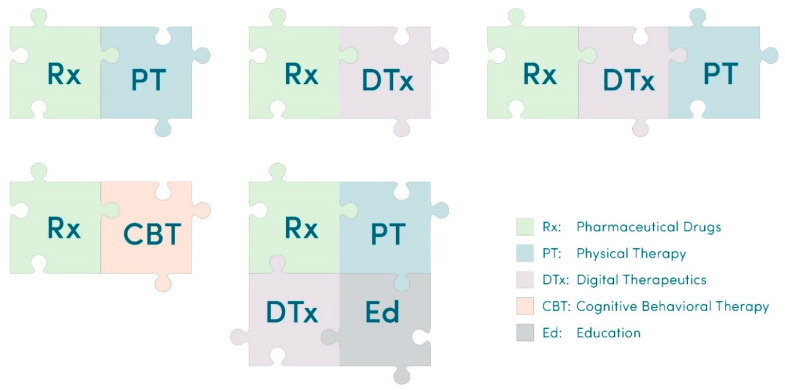
Diverse combinations of multimodal therapies enable personalized medicine for CLBP.

Figure 6 exemplifies how DTx can expand a repertoire of combination therapies for CLBP. Because software is versatile with respect to diverse digital content, e.g., videos with physical exercise (e.g., PT), interactive education, behavioral therapies (e.g., CBT), medication management (reminders and monitoring), activity tracking, motivational interviewing, mindfulness meditation, etc., it is perfectly positioned to integrate multiple therapeutic modalities. Patient education is an “active ingredient” that shows promise in treating CLBP [57,122,123,124,125]. As exemplified by RelieVRx and the Kaia Back Pain app, DTx are promising tools to integrate pharmacotherapies with patient education and PT (Figure 6).

**Figure 7:**
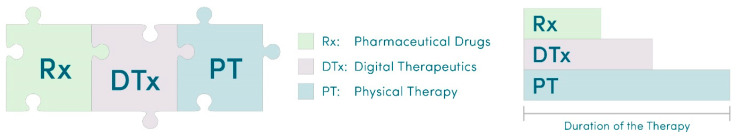
An example of the multimodal treatment of CLBP comprising analgesics, DTx and physical therapy. The duration of DTx and PT treatments can extend beyond the initial use of analgesics to reduce pain.

Opportunities for DTx to be integrated with pharmacotherapies as drug + digital combination therapies have been previously described for chronic pain and epilepsy [33,126,127]. Since digital health technologies can support medication management [128], digital therapeutics can help CLBP patients to support medication dosing, adherence and analgesic tapering while simultaneously providing other therapeutic modalities (e.g., CBT, mindfulness meditation, relaxation) and PT. After the duration of a DTx-based therapy, CLBP patients can continue PT and other types of physical activities (yoga, Pilates, etc.). Such “seamless” transitions during a multimodal therapy are shown in Figure 7. Eventually, patient education and physical exercises delivered via DTx and PT may result in developing self-management skills and habits that can reduce the risk of recurrence of CLBP.

## 5. Developing Exposome-Responsive DTx by Expanding Treatment Modalities

The exposome encompasses health-related environmental and lifestyle factors, and can be defined as “an integrated function of exposure on our body including what we eat and do, our experiences, and where we live and work” [129]. “The exposome concept strives to capture the diversity and range of exposures, including synthetic chemicals, dietary constituents, psychosocial stressors, and physical factors, as well as their corresponding biological responses.” [129]. Given the diversity of environmental, lifestyle and socioeconomic factors, and gene-environment interactions that influence both the cause and treatment of CLBP [14,18,130,131], it is apparent that DTx may address challenges to create personalized multimodal therapies tailored to the exposome of an individual patient.

**Figure 8:**
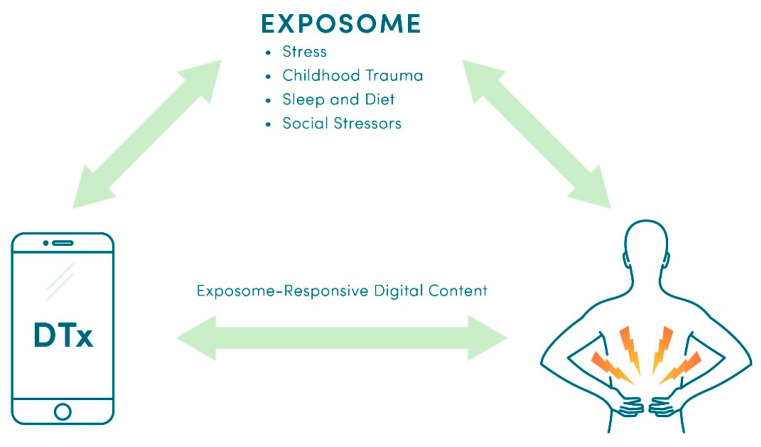
Exposome-responsive digital therapeutics delivering just-in-time adaptive interventions (JITAI) adjusted for a patient’s real-time needs and circumstances.

Development of exposome-responsive DTx for CLBP is illustrated in Figure 8. Since both stress and adequate sleep are associated with CLBP [132,133,134,135], development and validation of wearables that can measure stress and sleep [136] can also advance the development of biofeedback-based digital interventions for CLBP. We recently described how DTx can accommodate multiple modalities to yield personalized drug + digital combination therapies (“precision metapharmacology”) for people living with chronic diseases, such as chronic pain, epilepsy, depression, and cancer [126]. This approach enables integration of pharmacotherapies with just-in-time adaptive interventions (JITAI), a behavior-change therapy that can adjust content based on a patient’s real-time needs and circumstances [137,138]. For CLPB patients, JITAI can address sedentary behaviors [139,140,141,142] and comorbid depression [143], while seamlessly integrating medication management and the precision delivery of non-pharmacological “active ingredients” (PT, CBT, mindfulness meditation, breathing exercises, education, etc.). Given that adverse childhood experiences (ACE) and trauma-associated mental disorders impact both chronic pain and pain management [144,145], it is noteworthy that DTx have an ability to adjust therapeutic content based on ACE scores and emotions (Figure 1B in [126]).

One of the benefits of digital health technologies intended to treat chronic medical conditions is their ability to integrate diverse “active ingredients”, and both, RelieVRx and the Kaia Back Pain app are absolute examples of multimodal digital therapies. Herein, we suggest additional opportunities to create even more personalized interventions for CLBP by harnessing music-induced analgesia [146,147,148,149,150,151] and creating a home environment fostering chronic pain self-management [152]. Music-based interventions for acute and chronic pain show promising clinical outcomes [147,149,151,153,154,155,156], whereas using a mobile app to deliver music for opioid-based analgesia was also reported [148,157]. Music can support pain management via emotional and cognitive regulation [158,159,160,161,162], anti-inflammatory mechanisms [163,164], and potentially via the music-dopamine-reward axis as well [165,166,167]. A prototype of adjunct DTx delivering music-based intervention and self-management to reduce seizures in epilepsy patients [168] illustrates similar opportunities for chronic pain patients.

## 6. DTx Bridge Precision Medicine and Public Health

DTx can not only improve precision medicine for CLPB but can contribute to public health by: (1) decreasing the use of opioid-based analgesia and thus reducing opioid use disorders, (2) improving health literacy via patient education, and (3) improving therapy outcomes and preventing recurrence of CLBP, hence decreasing the burden of CLBP on healthcare and economy. Digital interventions to support opioid tapering are under development [169,170,171]. To improve the treatment of opioid use disorder, it is noteworthy that an adjunct prescription DTx, reSET-O (developed by Pear Therapeutics), delivers CBT for patients who also receive buprenorphine [172,173,174]. reSET-O was shown to improve therapy outcomes [174], decrease treatment costs [175], as well as reduce hospital readmissions and healthcare resource utilization [176].

Another public health benefit of digital health interventions is their ability to prevent the development and recurrence of chronic pain [177,178,179]. For example, patients who experienced musculoskeletal pain (including back pain) for less than 12 weeks had significantly higher odds of preventing chronic pain when using Hinge Health’s digital intervention (app-guided physical exercises, education, and virtual consultations), as compared to nonparticipants [177]. Preventive interventions can be more effective when using artificial intelligence-based technologies that can identify patients at risk for developing CLBP [180]. Since a combination of health education and physical exercises can be effective in preventing non-specific back pain [181], DTx delivering these modalities can yield both therapeutic and preventive effects. Mobile apps are recognized as means to improve health literacy and preventive behaviors [182,183,184]. Promotion and implementation of lifestyle modifications via DTx may lead to the development of sustainable self-care practices that decrease the risk for recurrence of CLBP.

## 7. Challenges and Limitations

Despite rapid advances in the development of DTx, there are many challenges in the implementation of digital technologies into healthcare. Reimbursement, interoperability, cybersecurity, patient engagement, attrition rates, evolving regulatory requirements, emerging evidence for clinical effectiveness, and cost-effectiveness of digital interventions are only a few examples that impact the adoption rates and usage of DTx. Commercialization challenges for DTx are illustrated by a recent bankruptcy filing of Pear Therapeutics (April 2023), which was one of the pioneers in developing DTx in the U.S. Adoption rates and reimbursement for digital interventions vary from country to country and are more advanced in Europe (e.g., Germany) compared to the U.S. [185,186,187].

Legal, regulatory, and business barriers to the implementation of DTx into healthcare systems result from a stark contrast between a rapid progress in digital technologies and a slow adoption of innovative medical solutions into clinical practice [188,189]. It is noteworthy that in March 2023, the RelieVRx was categorized as durable medical equipment by the Centers of Medicare and Medicaid Services, facilitating reimbursement codes for a VR program for CLBP patients in the U.S. Advancing digital health educational programs in medical, pharmacy, and nursing schools will also accelerate usage rates of DTx in the future [190,191,192].

## 8. Conclusions

In this perspective article, we describe unique opportunities for DTx to provide personalized, multimodal treatments for CLBP. DTx intended to treat CLBP, such as RelieVRx and the Kaia Back Pain app, expand non-pharmacological treatment options beyond those currently recommended in the clinical practice guidelines described by the American College of Physicians. It is important to raise awareness among healthcare professionals and patients about DTx and their abilities to integrate non-pharmacological interventions with pharmacotherapies. For R&D communities and funding agencies, it is important to advance the development of personalized digital interventions and health education. For policymakers and healthcare systems, it is important to accelerate adoption rates for DTX for CLBP and other chronic diseases.

## Data Availability

No new data were created or analyzed in this study. Data sharing is not applicable to this article.
